# A Systematic Review of the Phytochemical Profile and Potential Medicinal Functions of *Codonopsis pilosula* in Cancer

**DOI:** 10.1002/fsn3.70054

**Published:** 2025-02-24

**Authors:** Haoran Fan, Chenxi Ren, Yining Feng, Lingyi Zhu, Aobo Yu, Tianzhu Guan

**Affiliations:** ^1^ College of Tourism and Culinary Science Yangzhou University Yangzhou Jiangsu China; ^2^ School of Food Science and Engineering Yangzhou University Yangzhou Jiangsu China; ^3^ Jiangsu Vocational College of Tourism Yangzhou China; ^4^ The Second Norman Bethune Hospital of Jilin University Changchun China

**Keywords:** anti‐cancer activity, *Codonopsis pilosula*, network pharmacology, quantum‐chemical calculations, traditional Chinese Medicine

## Abstract

As a valuable medicine and food homology plant suitable for people of all ages, *Codonopsis pilosula* has been used for dietary nourishment and medicinal purposes with high acceptance for a long history. Interest in the potential anticancer functions of *C. pilosula* has increased due to its numerous chemical constituents with diverse structures and extensive pharmacological activities. With the growing interest, *C. pilosula* ‐based antitumor traditional Chinese medicine (TCM) formulations are also considered as vital intervention strategy for cancer, which exhibit prospective antitumor potential with multiple targets, multiple signaling pathways, and less side effects in both experimental and epidemiological studies. However, the prospective molecular mechanisms and newly emerging research methods in cancer auxiliary regulation require further elaboration. Consequently, this review systematically presents the latest research progress and future prospect of *C. pilosula* and highlights current gaps in knowledge, which facilitate the great rejuvenation of *C. pilosula* for the long‐term therapy use of tumor. Remarkably, with the gathering of the findings of biological evaluation, combinations with network computing approaches, such as network pharmacology, molecular docking, and quantum‐chemical calculations, this review is expected to provide theoretical support and open further research perspectives on *C. pilosula* in biological function and potential clinical efficacy.

## Graphical abstract

*Codonopsis pilosula* may serve as a potential treasure trove for pharmaceuticals, functional foods, and cosmetic additives in the future. The available evidence warrants further research into the possible role of *C. pilosula* in preventing human carcinogenesis. This review provides theoretical research and references for profound systematic testing and development in the use of *C. pilosula* in the fields of food and medicine.

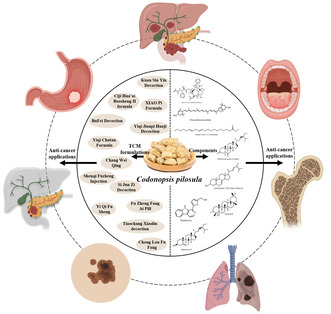

## 1 Introduction

*Codonopsis pilosula* , a traditional Chinese herb also known as Dangshen, has been used for centuries in folk medicine owing to its numerous health benefits (Luan et al. 2021). *C. pilosula* belongs to Campanulaceae and is mainly distributed throughout East, Central, and South Asia. Extensive research on the phytochemical and bioactive components of *C. pilosula* reveals its potential anti‐inflammatory, antioxidant, and cortisol‐regulating activities, which are responsible for its biological function, potential clinical efficacy and nourishing properties (Li et al. 2021; Zou et al. 2020b). Moreover, *C. pilosula* has been studied for its beneficial impacts on neurology, depression, and sleep disorders. The studies of medicinal plants within traditional Chinese medicine (TCM) are of great significance as they offer potential solutions to various health issues, and align with the current scientific pursuit of natural, sustainable, and effective health‐promoting agents. *C. pilosula* , as a medicine‐food homologous substance which can be used both as common food for daily consumption to provide nutrients and as a medicinal ingredient to help maintain health or relieve certain ailments based on the TCM theories, is of significant importance (Ren et al. 2024). TCM practitioners often incorporate *C. pilosula* in cancer auxiliary regulation by considering the holistic concept of syndrome differentiation, which not only inherits the wisdom of TCM but also aligns with the long‐term strategy of deeply exploring the multiple values of food and medicine homology (Chung et al. 2016; Matos et al. 2021).

Cancers characterized by abnormal cell growth remain a major public health concern in China, as about 3.065 million cases were reported in 2012, accounting for a quarter of global cancer‐related deaths (Sun et al. 2020). Conventional cancer interventions often come with serious side effects that can weaken the prognosis of patients. As a result, there have been growing interests in utilizing *C. pilosula* in personalized prescriptions to avoid the toxic side effects associated with chemotherapy, thus improving patient outcomes (Chen, Cheng, et al. 2021). The studies on the safety and effectiveness of *C. pilosula* in alleviating cancer‐related symptoms and side effects have yielded inconsistent findings (Liu et al. 2025). However, recent evidence implies combining multiple study designs through systematic reviews may be a promising way to evaluate the potential benefits of *C. pilosula* in auxiliary regulating cancer (Gao et al. 2018).

Renowned for its medicinal properties, *C. pilosula* has emerged as a popular complementary auxiliary regulation in cancer care. The multi‐bioactive effects of its active ingredients, such as polysaccharides, proteins, active peptides, lipids, terpenoids, and alkaloids, are believed to contribute significantly to its potential in combating cancers and auxiliary regulating associated symptoms. In China, where the prevalence and severity of cancers pose a substantial public health challenge, there exists a notable gap in the precise identification of the functional components within *C. pilosula* and related formulas in cancer auxiliary regulation. Current research has not comprehensively explored how these components exert their anti‐cancer activities and alleviate cancer‐related symptoms. Our review aims to fill this gap by systematically summarizing and assessing the reported anti‐cancer phytochemical and bioactive functions of *C. pilosula* and related formulas, thus offering data that is not only conducive to its common use in TCM but also provides a solid foundation for in‐depth scientific research.

## 2 Phytochemical Profiling of *C. pilosula*

**FIGURE 1:**
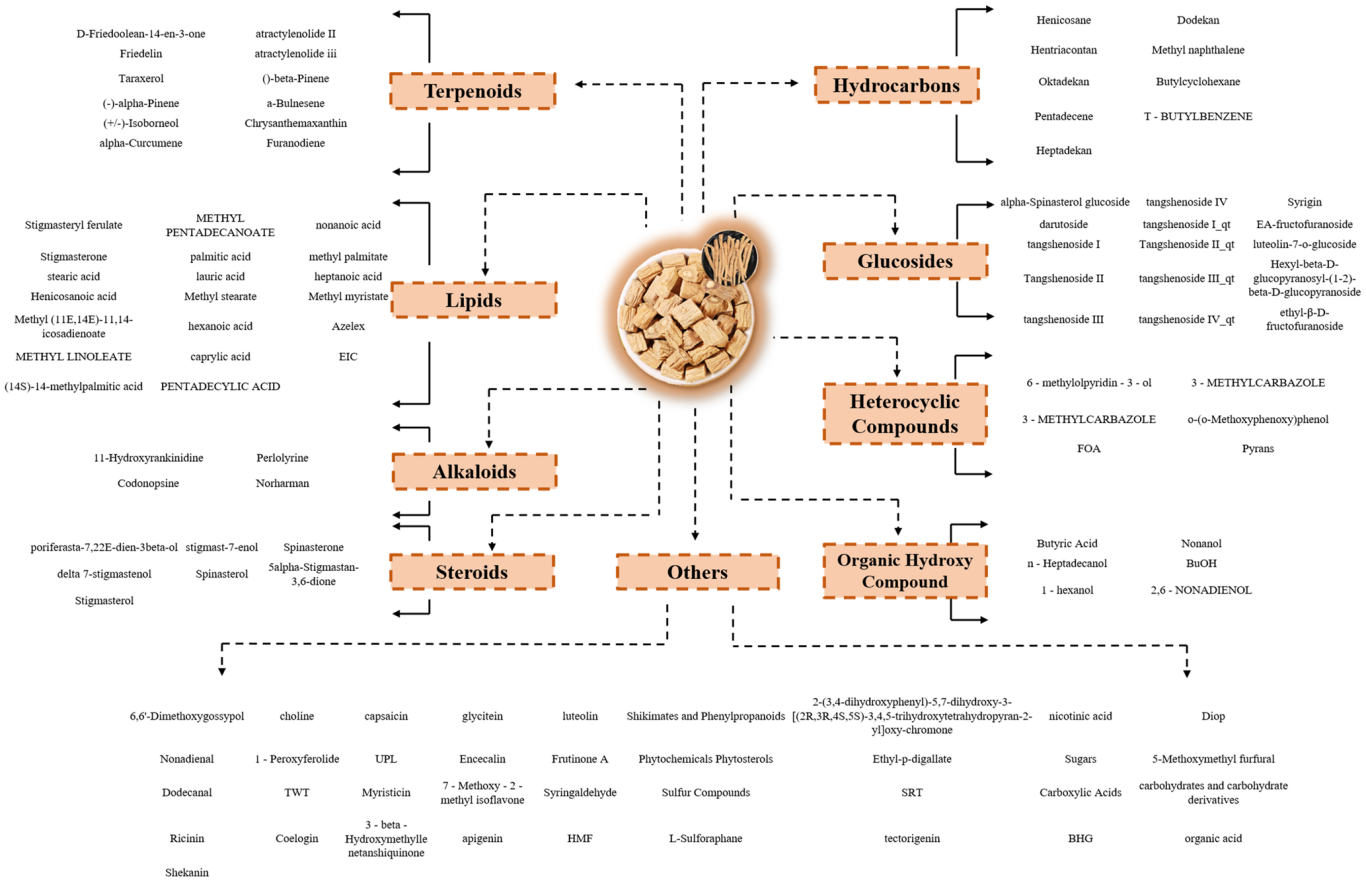
Classification and summarization of active ingredients from *Codonopsis pilosula*.

As a precious treasure of food‐medicine homology in China, *C. pilosula* has merits in auxiliary regulating cancer owing to the abundant nutritional components and physiologically‐active compounds. Thus, in this study “ *C. pilosula* ” and “*Dangshen*” were used as keywords to acquire component information from the System Pharmacology Database and Analysis Platform. Over the past decades worldwide, numerous research on chemical constituents isolated from *C. pilosula* has been reported and their structural formulas have been summarized completely (Figure 1), which have reference values. The reported anticancer constituents of *C. pilosula* extracts are diverse, covering both small‐molecule and complex polysaccharides, bidesmosidic triterpenoid saponins (e.g., lancemasides A–B), pyrrolidine alkaloids, xanthone derivatives (e.g., coxanthone B, swertiperenine), and polyacetylene glycosides (e.g., lobetyolin).

**FIGURE 2:**
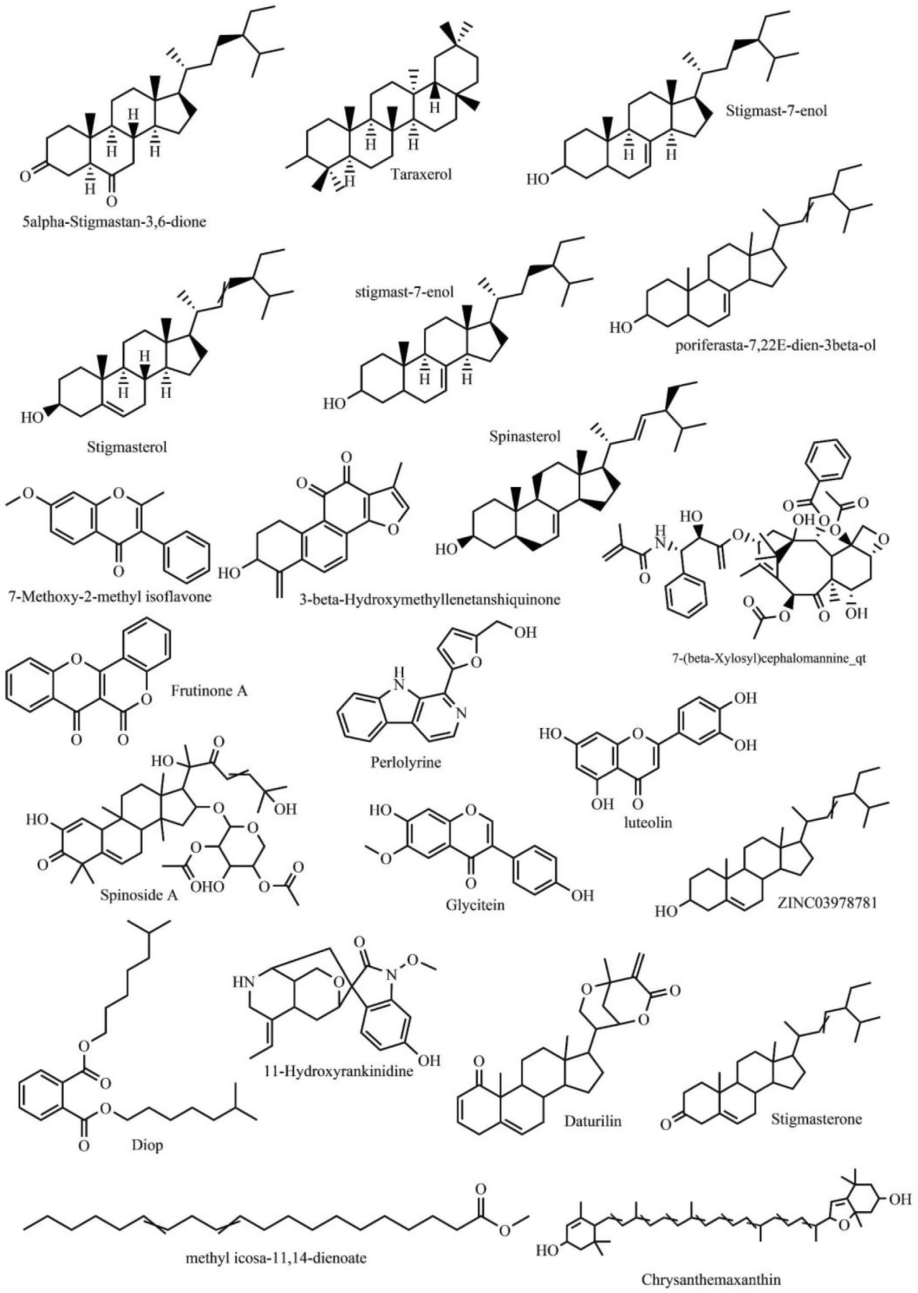
Chemical structures of active constituent (OB ≥ 30%, DL ≥ 0.18) from *Codonopsis pilosula*.

As studies imply various chemical constituents improve the effects of *C. pilosula* , the absorption, distribution, metabolism, and excretion (ADME) properties in different extract fractions can also significantly affect the structure and bioactivity of bioactive components from *C. pilosula* (Vugmeyster et al. 2012). The in silico integrative ADME model under TCMSP is used in pharmaceutical studies. Here, two related ADME standards (oral bioavailability [OB] and drug likeness [DL]) were adopted to recognize potential bioactive compounds. OB reflects the rate and degree at which active compounds or groups of the drug are absorbed in systemic circulation, and mainly assesses if the drug can be developed. Basically, an OB value of ≥ 30% is considered high. Also, the DL values of all herbal medicines are obtained by calculating the Tanimoto coefficient. Generally, DL ≥ 0.18 means high drug likeness. Thus, the compounds with OB ≥ 30% and DL ≥ 0.18 that meet the criteria from TCMSP (duplicates removed) remain as the candidate compounds for further research (Figure 2) (Li et al. 2022; Lu et al. 2022). The specific parameters are shown in Table 1.

**TABLE 1:** Chemical structures of active constituents (OB ≥ 30%, DL ≥ 0.18) from *Codonopsis pilosula* .

Molecular name	MW	ALOGP	Hacc	OB (%)	Caco‐2	BBB	DL
5alpha‐Stigmastan −3,6‐dione	428.77	6.66	2	33.12	0.9	0.47	0.79
Daturilin	436.64	4.34	4	50.37	0.61	0.06	0.77
Taraxerol	426.8	7.3	1	38.4	1.37	1.18	0.77
Stigmast‐7‐enol	410.75	7.31	1	45.4	1.49	1.26	0.76
Stigmasterol	412.77	7.64	1	43.83	1.44	1	0.76
ZINC03978781	412.77	7.64	1	43.83	1.32	0.96	0.76
Poriferasta‐722e‐Dien‐3beta‐Ol	412.77	7.64	1	42.98	1.45	1.11	0.76
Spinasterol	412.77	7.64	1	42.98	1.44	1.04	0.76
Stigmast‐7‐Enol	414.79	8.08	1	37.42	1.39	1.04	0.75
11‐Hydroxyrankinidine	356.46	1.04	6	40	0.29	−0.19	0.66
Chrysanthemaxanthin	584.96	8.24	3	38.72	0.51	−0.98	0.58
3‐beta‐Hydroxymethyllenetanshiquinone	294.32	3.16	4	32.16	0.38	−0.48	0.41
Spinoside A	716.95	2.91	12	39.97	−1.02	−1.76	0.4
Diop	390.62	7.44	4	43.59	0.79	0.26	0.39
Frutinone A	264.24	2.7	4	65.9	0.89	0.46	0.34
7‐(beta‐Xylosyl)cephalomannine_qt	830.02	3.21	14	38.33	−0.87	−1.59	0.29
Perlolyrine	264.3	3.2	3	65.95	0.88	0.15	0.27
Luteolin	286.25	2.07	6	36.16	0.19	−0.84	0.25
Glycitein	284.28	2.32	5	50.48	0.56	−0.29	0.24
Methyl Icosa‐11,14‐Dienoate	322.59	7.55	2	39.67	1.47	1.1	0.23
7‐Methoxy‐2‐methyl isoflavone	266.31	3.36	3	42.56	1.16	0.56	0.2

As indicated above, the constituents of *C. pilosula* contributing to its pharmacological effects can be classified into polysaccharides, proteins, active peptides, lipids, terpenoids, and alkaloids. Recently, with the advancement of emerging extraction techniques, an increasing number of active components in *C. pilosula* have been successfully extracted and identified. These active components have sparked great interest in food science and biology. Exploration and characterization of these components will potentially uncover new applications for auxiliary regulation on cancers and contribute to the development of natural‐based means for it.

Due to their significant bioactivities and pharmaceutical potency, these active components (polysaccharides, proteins, active peptides, lipids, terpenoids, and alkaloids) with various pharmacological properties such as anti‐cancer, neuroprotective and immunomodulatory effects are critical in *C. pilosula* . Despite the growing interest in *C. pilosula* and its active components, there is still a lack of comprehensive understanding of their auxiliary regulating potential on cancers. Further in‐depth research and exploration are needed to fully unravel the mechanisms underlying the anti‐cancer activities of *C. pilosula* and its active components.

## 3 The Innovative Data Mining and Applications of *C. pilosula* and Related Formulas in Cancer Auxiliary Regulation

It is well known that the composition of *C. pilosula* and related formulas, which contains hundreds of compounds, is extraordinarily complex. Compared with a single compound, *C. pilosula* and related formulas show synergistic auxiliary regulating effects, which involve multiple targets and multiple signal pathways, regulating the activities and expression of a series of proteins. Therefore, TCM formulations play an indispensable role in the auxiliary regulation of multifactorial chronic diseases including cancer. Although many recipes of medicated diets have been developed for a long time, the hundreds of chemicals in the formulation and their interactions are not well‐known. However, some major hurdles in theoretical research and practical use need to be handled. Emerging experimental technologies, such as multi‐omics (proteomics, metabolomics, single‐cell sequencing, and transcriptomics), high‐throughput screening, and gene editing have accelerated the development of pharmacological research. The accumulation of experimental data related to *C. pilosula* also reveals that investigation into anti‐cancer effects of *C. pilosula* reveals the intersection of multidisciplinary trends, including the profound integration of computational, testing and clinical methods.

**FIGURE 3:**
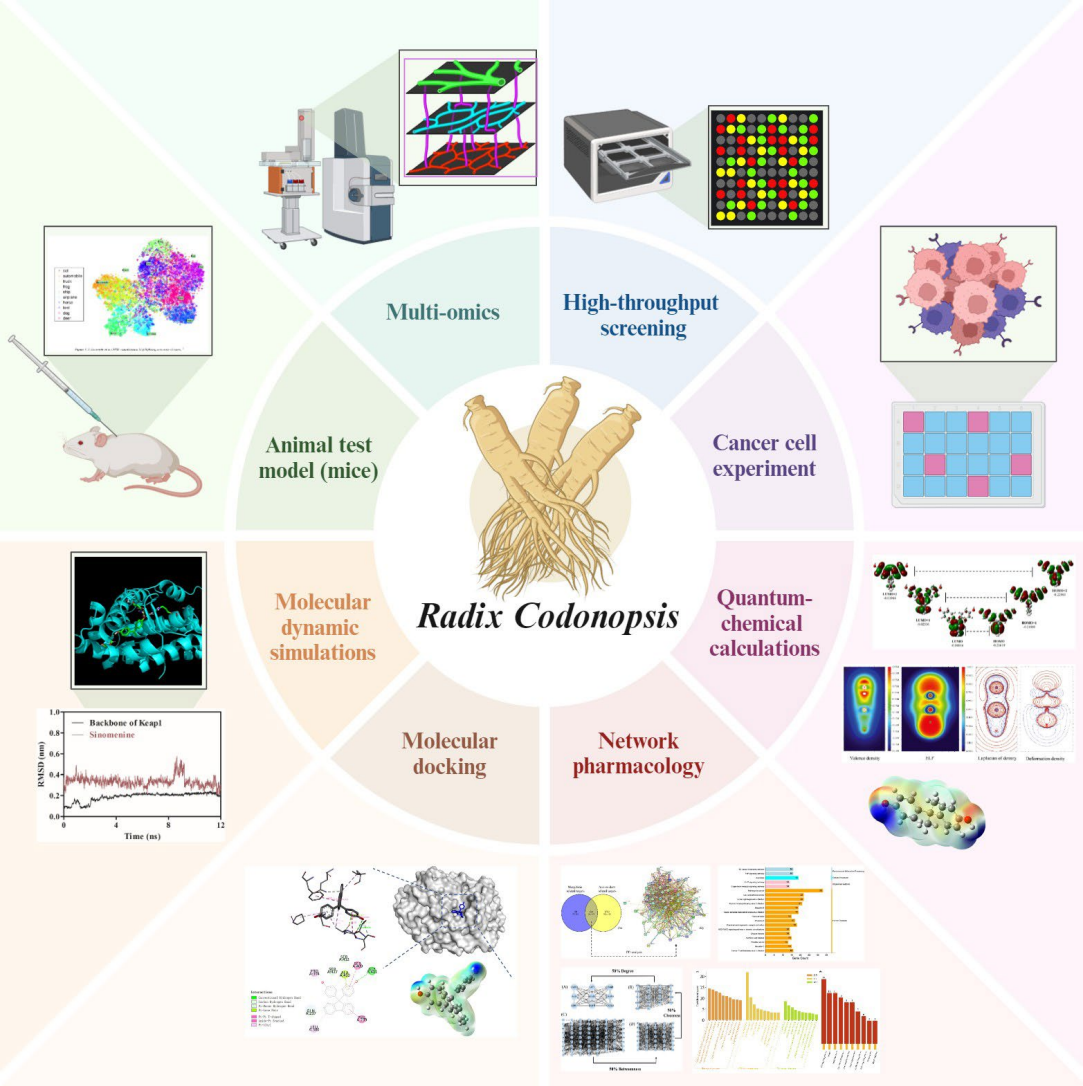
Integration strategy of *Codonopsis pilosula* study.

With the rise of interdisciplinary fields such as bioinformatics and system biology, the new‐generation research schemes for exploring the interactions between *C. pilosula* and cancer auxiliary regulation have gradually shifted from separate research to systematic investigation. From the holistic view of TCM, *C. pilosula* is typical of multi‐target and multi‐pathway owing to the complexity of chemical components in systematic and thorough disease auxiliary regulation. Hence, investigation into the specific active components and potential mechanisms is difficult (Fan et al. 2024). As a novel method, network pharmacology allows for systematic prediction of potential mechanisms between known targets and is suitable for the research on multi‐component complexes (Li, Gao, et al. 2024). Based on protein, gene, drug, and disease databases, this approach completely integrates targeted pharmacology with computational biology and bioinformatics to uncover the “target‐ingredient‐pathway” interactions, which accords with the holistic part of Chinese medical diet theories (Guan et al. 2023). Additionally, molecular docking, molecular dynamic simulations, and quantum‐chemical calculations were further used to serve as theoretical guidance for active compound‐target complexes (Zhang, Zhao, Xue, et al. 2024). All the calculated methods shall focus on the energetic and geometrical indices in the optimal conformations of the molecules, which contain rich computer‐based proofs and theoretical interpretations for ingredient‐target interactions. These new computer‐based approaches (Figure 3) were applied to various formulas, such as Zhishi Rhubarb Soup, Peony and Licorice and Aconite Decoction, and Shennao Fuyuan Tang (Guan et al. 2023; Zhang, Zhao, Li, et al. 2024).

## 4 Mechanisms of *C. pilosula* and *C. pilosula* ‐Based TCM Formulations in Auxiliary Regulating Cancer

Although surgery, chemotherapy and radiotherapy are the mainstay of cancer therapy, TCM can reduce cancer therapy‐caused toxicity and is a popular complementary and alternative medicine (CAM) in China. With thousands of years of medical practice, valuable experience in TCM for preventive intervention has been gathered, which is one core concept of TCM. The classic formulas of many traditional prescriptions in clinic have been revised to minimally decrease the side effects of surgery, radiation and chemotherapy, and to improve immune function and enhance survival rates. Many *C. pilosula* ‐based TCM formulations with outstanding curative effects (e.g., Sijunzi decoction and Xiangshaliujunzi decoction) have been used for the auxiliary regulation of various diseases with different medical emphasis subject to the altered symptoms for years and are still in use. We have summarized studies involving the mechanisms of *C. pilosula* and *C. pilosula* ‐based TCM formulations in auxiliary regulation of various cancers. Among the active components of *C. pilosula* , the polysaccharides have been the main focus as they play a crucial role in the anti‐cancer process. Their unique chemical structures and biological activities are believed to be closely associated with the anti‐cancer effects observed in these studies.

### 4.1 Application in Lung Cancer

Lung cancer is among the most harmful human diseases and has the highest incidence worldwide. The onset and progression of lung cancer involve several intricate pathological processes. It is also prone to metastasis, which can lead to drastic reversal in a clinical setting and affect the liver, brain, bone, and lymph nodes. Numerous studies have demonstrated that *C. pilosula* and its prescriptions exhibit unique advantages in the auxiliary regulation of lung cancer and possess great potential in combating lung cancer.

As a potential solution for drug resistance and side effects, the intake of *C. pilosula* ‐based compounds plays a key role in protecting liver cells and reducing the incidence rate of liver cancer. Yang et al. (2013) first isolated and characterized a pectic polysaccharide named *C. pilosula* polysaccharide 1b (CPP1b) from *C. pilosula* . Combined with sugar and spectroscopic analyses, it was found that CPP1b significantly inhibited the proliferation of human lung adenocarcinoma A549 cells in a time‐ and dose‐dependent way. On this basis, Chen et al. (2014) synthesized selenium–CPP1b (sCPP1b) via an HNO_3_^−^Na_2_SeO_3_ method. Astonishingly, sCPP1b exhibited more vigorous cytotoxicity on A549, HeLa, and BGC‐823 cells than CPP1b, indicating that sCPP1b is a potential antitumor agent candidate. Furthermore, Wang confirmed *C. pilosula* exerted antibacterial activities and improved the lung dysbiosis of tumor‐bearing mice via inducing lung cancer cell apoptosis and improving lung dysfunction by deactivating the Ras/PI3K/AKT pathway. This finding suggests that *C. pilosula* may be a candidate for auxiliary regulating lung cancer in humans (Wang et al. 2022). To sum up, both the active components such as polysaccharides in *C. pilosula* itself and the chemically‐modified products have significant effects on the inhibition of lung cancer cells. Moreover, they also play active roles in improving the body functions and microenvironment related to lung cancer, further demonstrating the crucial value of *C. pilosula* in the auxiliary regulation of lung cancer (Huang et al. 2024).

Liu et al. (2018) demonstrated that BuFei Decoction inhibited representative non‐small cell lung cancer (NSCLC) cell lines and a xenograft model by inhibiting apurinic/apyrimidinic endonuclease 1. These results suggest that Bu‐Fei decoction may be a promising auxiliary regulation for NSCLC. Wang et al. (2015) found Yiqi Chutan Formula may exert anti‐tumor effects via regulating prolyl 4‐hydroxylase beta‐polypeptide in endoplasmic reticulum to inhibit the occurrence and procession of Lewis lung carcinoma, which may be a critical anti‐cancer mechanism of TCM. Liu et al. (2014) found Fu Zheng Fang Ai Pill combined with cyclophosphamide remarkably decelerated the growth and metastasis of C57BL/6 mice subcutaneously injected with Lewis lung cancer cells through inhibiting SOCS/JAK–STAT pathway and inflammatory cytokine responses. This study, featured by moderate long‐lasting efficacy and few side effects of Fuzheng Fangai pill, enriches the scientific connotation and brings one new direction. Current observations indicate that Kuan Sin Yin Decoction, as a compound formula, may immunomodulate to restrict tumor growth and act as a complementary approach for cancer auxiliary regulation. Li et al. (2014) confirmed that Kuan‐Sin‐Yin suppressed lung cancer proliferation without apparent toxicity in vivo and in vitro, because it was cytostatic to cancer cells. These studies fully demonstrate that various *C. pilosula* ‐based prescriptions, such as Bu‐Fei decoction, Yiqi Chutan Formula, Fu Zheng Fang Ai Pill, and Kuan Sin Yin Decoction, starting from different action mechanisms, including the regulation of relevant enzymes, cellular pathways, and immunomodulation, have positive significance for the auxiliary regulation of lung cancer and the inhibition of tumor growth. They provide abundant options for the comprehensive auxiliary regulation of lung cancer and reflect the broad application prospects of TCM formulations in the field of anti‐cancer.

### 4.2 Application in Liver Cancer

Liver cancer is one of the most common causes of cancer‐induced death worldwide, with a global incidence rate of 43.7%. Despite the considerable headway in new prevention, screening, diagnosis and treatment technologies in recent years, its incidence rate and mortality are still rising owing to its strong growth, proliferation, adhesion, invasion and metastasis capabilities. The routine treatment at the late stage of liver cancer is chemotherapy, which kills tumor cells while destroying normal cells.

Nowadays, several experimental results theoretically underlie the auxiliary regulation of liver cancer with *C. pilosula* . Zhang et al. (2019) observed that *C. pilosula* polysaccharides (CPP) presented an inhibitory effect on liver cancer via weakening the invasion and metastasis of HepG2 cells in vitro, delaying epithelial–mesenchymal transition (EMT) in HepG2 cells, and inhibiting the expression of β catenin/T‐cell factor 4 protein. Ko et al. (2019) also proved via in vitro experiments that CPP can effectively inhibit HepG2 cell growth. Both the genomic approach of microarrays and qRT‐PCR validation revealed that GDF15 and HMOX1 may be potential biomarkers in hepatic cells. Li, Yang, et al. (2024) revealed the detailed anti‐tumor mechanisms of CPP using network pharmacology and transcriptome sequencing in vitro and in vivo. Like network pharmacology analysis, *C. pilosula* presented promising inhibition on liver cancer growth via suppressing the expressions of CDK1/PDK1/β‐catenin signaling factors, limiting cell EMT and inducing cell apoptosis. As for Se nanoparticles (SeNPs) stabilized by inulin fructans from *C. pilosula* , biological tests showed that the SeNPs stabilized by an inulin fructan from *C. pilosula* were selectively cytotoxic to Huh‐7 and HepG2 cells, with strong anti‐proliferative and pro‐apoptotic activities. These results provide insights into the potential role in auxiliary regulation of *C. pilosula* in liver cancer (Hu et al. 2022). Yu et al. (2023) found luteolin markedly inhibited the proliferation and migration of liver cancer based on the network pharmacology. These findings indicate luteolin of CPP remarkably resisted the phosphorylation of MAPK‐JNK and Akt (Thr308) and then led to upregulation of ESR1. Taken together, multiple experimental results provide theoretical support for *C. pilosula* in the auxiliary regulation of liver cancer. The various chemical components of *C. pilosula* exert anti‐liver cancer effects by acting on the relevant mechanisms of liver cancer cells. Therefore, *C. pilosula* , as an option for auxiliary regulation of liver cancer, holds great potential and relatively high safety.

The potential of *C. pilosula* in the auxiliary regulation of liver cancer is remarkable. The numerous studies mentioned above have provided theoretical basis for the application of *C. pilosula* in liver cancer auxiliary regulation from different perspectives. From the inhibition of the proliferation and migration of cancer cells to the impacts on relevant signaling pathways and biomarkers, the medicinal value of *C. pilosula* has been fully demonstrated. On this basis, the research on prescriptions containing *C. pilosula* has brought new hope for liver cancer auxiliary regulation. Fu et al. (2019) demonstrated that Ciji Hua'ai Baosheng II formula exerted significant effects in a mouse model, into which an H22 liver cancer cell suspension was injected. Specifically, this formula led to a remarkable reduction in tumor volume and anti‐apoptotic protein expression. Meanwhile, it increased the expression of pro‐apoptotic protein and induced apoptosis of tumor cells. At present, in the face of the severe challenge of combating liver cancer, the number of truly effective anti‐liver cancer prescriptions remains limited. The relevant research is still in its infancy, and a great deal of exploratory work awaits to be done. More effective anti‐liver cancer prescriptions still need to be developed.

### 4.3 Application in Colorectal Cancer

The occurrence and mortality rates of colorectal cancer (CRC) have been progressively increasing for years in China, particularly in people aged 65–79 years. The TCM for CRC auxiliary regulation, which overcomes the restriction of radiotherapy, chemotherapy and surgery, has crucial research significance. However, although the auxiliary regulation effects of *C. pilosula* and related prescriptions for CRC have been confirmed, there are few studies on their expected auxiliary regulation effects and molecular mechanisms.

Wang et al. (2010) verified through in vitro cell experiments that the n‐butanol fraction (BF) of *C. pilosula* can induce G0/G1 phase arrest and apoptosis of HT‐29 cells in a dose‐and time‐dependent manner, thus showing anti‐CRC effects. Specifically, this inhibition relates to intracellular ROS generation and polyamine depletion. There was enhanced caspase‐3 and p53 expression, increased Bax/Bcl‐2 ratio, and reduced survivin expression in BF‐treated HT‐29 cells. *C. pilosula* can remarkably restrain the multiplication of CRC. Collectively, when combined with human tumor necrosis factor‐related apoptosis‐inducing ligand TRAIL, it significantly inhibits the proliferation of HCT116 cells (Zou et al. 2020a). The n‐butanol fraction of *C. pilosula* can induce cell‐cycle arrest and apoptosis of cancer cells to resist CRC, which involves the alteration of intracellular substances and the regulation of protein expression. These preliminary research findings on the anti‐CRC effects of *C. pilosula* have provided important clues for further exploring its application potential in the auxiliary regulation of CRC. However, it is still necessary to further explore its mechanism of action in the complex in vivo environment and the possibility of combination with other herbs.

Zhou et al. (2019) elucidated the underlying mechanism of modified Si Jun Zi Decoction against multiple malignancies via a nude mouse model with splenic transplantation of colon cancer cells. The modified Si Jun Zi Decoction increased the survival rate and reduced CRC liver metastasis by stimulating the innate immune system. This finding offers a complementary and alternative auxiliary regulation approach for CRC. Chang Wei Qing is a Chinese herbal recipe with clinical efficacy proposed by the famous TCM practitioner Fan Zhong Ze. Wan et al. (2019) demonstrated that high‐concentration Chang Wei Qing remarkably decreased tumor size and quantity, and inhibited NF‐κB and STAT3 pathways to retard colitis‐related tumor progression, which mainly contributed to the potential benefits of Chang Wei Qing in drug‐caused colitis‐related cancer auxiliary regulation. Deng et al. (2013) conducted chemical characterization on Yi‐Qi‐Fu‐Sheng with *C. pilosula* as the main component (which also contains *Atractylodes macrocephala*, *Poria*, *Radix glycytthizae*, *Myristica fragrans* , and *Fiveleaf akebia fruits*) and determined its anti‐tumor effect in CRC. Yi‐Qi‐Fu‐Sheng induced apoptosis in vitro and in vivo, inhibited CRC cell proliferation and the expression of ERK1/2‐dependent MMP‐2/9. Chen, Zhang, et al. (2021) highlighted that TiaochangXiaoliu decoction significantly decreased the tumor number and inflammatory cell infiltration in CRC mice. The decoction markedly reduced the serum expression of cytokines, and increased the contents of T lymphocyte subset cells in the plasma of mice with azomethane/dextran sulfate sodium‐induced CRC. The decoction is a potential strategy for CRC targeting auxiliary regulation. The above studies have fully demonstrated the unique advantages and potentials of different TCM formulations in the auxiliary regulation of CRC, laying a solid foundation for further research on the synergistic effects of TCM in the comprehensive auxiliary regulation of CRC, optimizing formulations, and developing new strategies for auxiliary regulation.

### 4.4 Application in Breast Cancer

Globally, the incidence of breast cancer is becoming increasingly severe. Although Western medicine is important in the treatment of breast cancer, it presents numerous challenges. On the one hand, Western medicine is costly, bringing a heavy financial burden to patients' families. On the other hand, it shows adverse side effects, affecting patients' quality of life and even physical functions. Therefore, it is extremely urgent to explore herbs and prescriptions with anti‐breast cancer activities.

The experiments conducted by Chen et al. (2018) demonstrated that the exopolysaccharide derived from the endophyte of *C. pilosula* was capable of inducing macrophage activation and polarization, facilitating the production of TNF‐α and nitric oxide, and enhancing macrophage infiltration. Concurrently, it inhibited the migration of breast cancer cells (BT549 and MDA‐MB‐231). Such an action could result in cell‐cycle arrest at the S‐phase and trigger apoptosis in cancer cells. Moreover, it could impact the orientation and positioning of the spindle, thereby manifesting anti‐breast cancer effects. Wang et al. (2014) demonstrated that Codonolactone can inhibit the invasion and migration of breast cancer cells (MDA‐MB‐231 and MDA‐MB‐157), significantly suppressing the breast cancer in vivo. The anti‐breast cancer mechanism of Codonolactone is associated with the inhibition of the activity and expression of matrix metalloproteinases (MMP‐9 and MMP‐13). By downregulating the activity of the Runx2 transcription factor and restraining the binding of Runx2 to the MMP‐13 promoter sequence, it exhibits anti‐breast cancer activity. Substances derived from *C. pilosula* play significant roles in the auxiliary regulation of breast cancer. Their anti‐breast cancer mechanisms are associated with influencing the functions of immune cells, the cell cycle and apoptosis of cancer cells, inhibiting the migration and invasion of cancer cells, as well as regulating the activities and expressions of relevant enzymes and transcription factors, providing directions for further research.

Zheng et al. (2020) found that XIAOPI formula inhibited breast cancer cell proliferation and metastasis via regulating TAMs/CXCL1 signaling and decreasing hematopoietic stem/progenitor cells (HSPC) level. In line with in vivo studies, XIAOPI formula also suppressed premetastatic niche (PMN) formation by inhibiting HSPC recruitment and myeloid‐derived suppressor cells (MDSCs) gathering in lung tissues. Liu et al. (2020) combined network pharmacology with molecular docking to explore the underlying mechanisms by which Shenqi Fuzheng injection, composed of the extracts of *C. pilosula* and *Radix astragali*, assists in regulating breast cancer. Among them, the results of network analysis demonstrated that the high‐affinity binding between bioactive compounds and their corresponding targets might synergistically contribute to the auxiliary regulation effects against breast cancer. Moreover, the core targets were enriched in multi‐biological pathways, such as nitrogen metabolism and HIF‐1 signaling pathway, which hold promise for the complete auxiliary regulation of breast cancer. It is worth emphasizing that *C. pilosula* , as one of the crucial components of Shenqi Fuzheng injection, plays an indispensable role in this underlying auxiliary regulation mechanism. The abovementioned research provides significant evidence for the exploration of the application value of *C. pilosula* in the field of breast cancer auxiliary regulation.

### 4.5 Application in Gastric Cancer

Gastric cancer is among the most frequent cancers globally, ranking the fifth in morbidity and the fourth in fatality among malignant tumors, according to the 2020 World Health Organization (WHO) Cancer Report (Arnold et al. 2020). Currently, the major treatment procedure of gastric cancer is still surgical resection plus chemotherapy, which will generate unavoidable severe side effects and treatment resistance (Joshi and Badgwell 2021). In contrast, TCM has numerous benefits in auxiliary regulating gastric cancer (Xu et al. 2022).

The advantages of TCM in auxiliary regulation of gastric cancer have been gradually demonstrated through various studies on *C. pilosula* . Tang et al. (2021) revealed the pharmacological mechanism of *C. pilosula* in auxiliary regulating gastric cancer using network pharmacology and molecular docking. Luteolin and cryptotanshinone, the main active compounds of *C. pilosula* in auxiliary regulating gastric cancer, were involved in the auxiliary regulation through a multi‐target and multi‐pathway route. Based on the multi‐omics analysis and serum pharmacology, He et al. (2022) found *C. pilosula* resists gastric precancerous lesions by relieving gastritis injury and selectively inhibiting the division of gastric cancer cells rather than normal cells. The abovementioned studies have shown that the active components in *C. pilosula* can exert anti‐gastric cancer effects through multi‐targets and multi‐pathways. Moreover, *C. pilosula* can combat precancerous lesions of gastric cancer. These findings not only provide new ideas for clarifying the mechanisms of complex systems such as TCM formulations but also offer auxiliary regulation options for resisting precancerous lesions of gastric cancer.

Moreover, these findings further emphasize the significance of exploring more TCM formulations containing *C. pilosula* in the auxiliary regulation of gastric cancer, as they may provide more options and potential breakthroughs in combating this formidable disease. Yiqi Jianpi Huaji Decoction is a TCM 12‐herb formula, in which *C. pilosula* is the principal ingredient. To probe the underlying molecular mechanism of this decoction, Li et al. (2015) demonstrated that the low‐dose formula alleviated the proliferation of human gastric cancer SGC7901/VCR cells, increased chemotherapeutic sensitivity, and reversed multiple drug resistance. Chong Lou Fu Fang, composed of *Fructus forsythiae*, *Rhizoma paridis*, and *C. pilosula* , is regarded as a potential adjuvant to chemotherapy. In vitro and in vivo results confirm that the combination of Chong Lou Fu Fang and chemotherapeutic agents can disturb cell cycle and inhibit human gastric cancer proliferation via synergistically downregulating genes associated with chemotherapeutic agent resistance (Liu et al. 2011). In summary, the studies on *C. pilosula* have shown its promising potential in different aspects of gastric cancer auxiliary regulation, from interfering with the development of precancerous lesions to affecting the auxiliary regulation of existing gastric cancer.

### 4.6 Application in Other Cancers

The abovementioned studies have demonstrated that *C. pilosula* and *C. pilosula* ‐based TCM formulations possess anti‐lung cancer, anti‐liver cancer, anti‐CRC, anti‐breast cancer, and anti‐gastric cancer effects. In addition to the auxiliary regulation effects on the aforementioned cancers, *C. pilosula* also exerts certain pharmacological effects on other cancers, including pancreatic ductal adenocarcinoma (PDAC), malignant melanoma, osteosarcoma (OS), and oral cancer.

PDAC, as a devastating disease, is attracting increasing attention because it is the fourth most common cause of disease‐associated mortality globally. Luan et al. (2018) systematically tested the possible effects of cordifoliketones A from *C. pilosula* on PDAC cells. The results showed cordifoliketones A inhibited viability, invasion and migration of PDAC cells via inducing apoptosis and disturbing apoptosis‐associated protein expressions in a dose‐dependent manner, indicating cordifoliketones A may be a potential candidate compound to prevent PDAC.

Malignant melanoma is a very invasive tumor. Lymphatic and hematogenous metastasis can happen early in tumor generation, leading to poor prognosis. The incidence and mortality of melanoma have risen remarkably recently, but options for auxiliary regulation, such as surgery and chemotherapy, are limited due to some side effects. CPP are attracting growing attention with their potential functions in auxiliary regulating chronic diseases (e.g., tumors) and regulating the immune system. Liu et al. (2021) demonstrated that CPP inhibited the proliferation of IL‐4‐induced M2‐like TAMs, and remarkably upregulated the mRNA levels of IL‐1/6, iNOS, and TNF‐a, increasing the repolarization of M2‐like to M1‐like TAMs.

As the most common primary bone tumor in children and adults, OS is a malignancy that mainly impacts long bones or other bones, and is featured by high metastatic and mortality rates, and poor prognosis. Gong et al. (2021) recognized 15 potential compounds and 48 core targets via network pharmacology analysis. GO and KEGG pathway enrichment indicated the anticancer effect of *C. pilosula* on OS through regulating potential auxiliary regulation targets, which are all essential mediators of enriched signaling pathways. These findings demonstrate the importance of understanding the potential auxiliary regulation mechanisms of *C. pilosula* against OS.

According to the WHO, oral cancer is among the most prevalent cancers worldwide (Huang et al. 2024). High‐risk behaviors such as smoking, excessive alcohol consumption, and betel‐nut chewing significantly increase the likelihood of developing oral cancer. Patients often experience severe pain, and their quality of life deteriorates due to difficulties in speaking, eating, and swallowing (Chuang et al. 2022). Shin et al. (2012) found the methanol extracts of *C. pilosula* inhibited growth and induced apoptosis by increasing Bak protein expression of HSC‐2 human oral cancer cells. It is indicated that the natural products from *C. pilosula* have non‐toxic auxiliary regulation potential against oral cancer.

**FIGURE 4:**
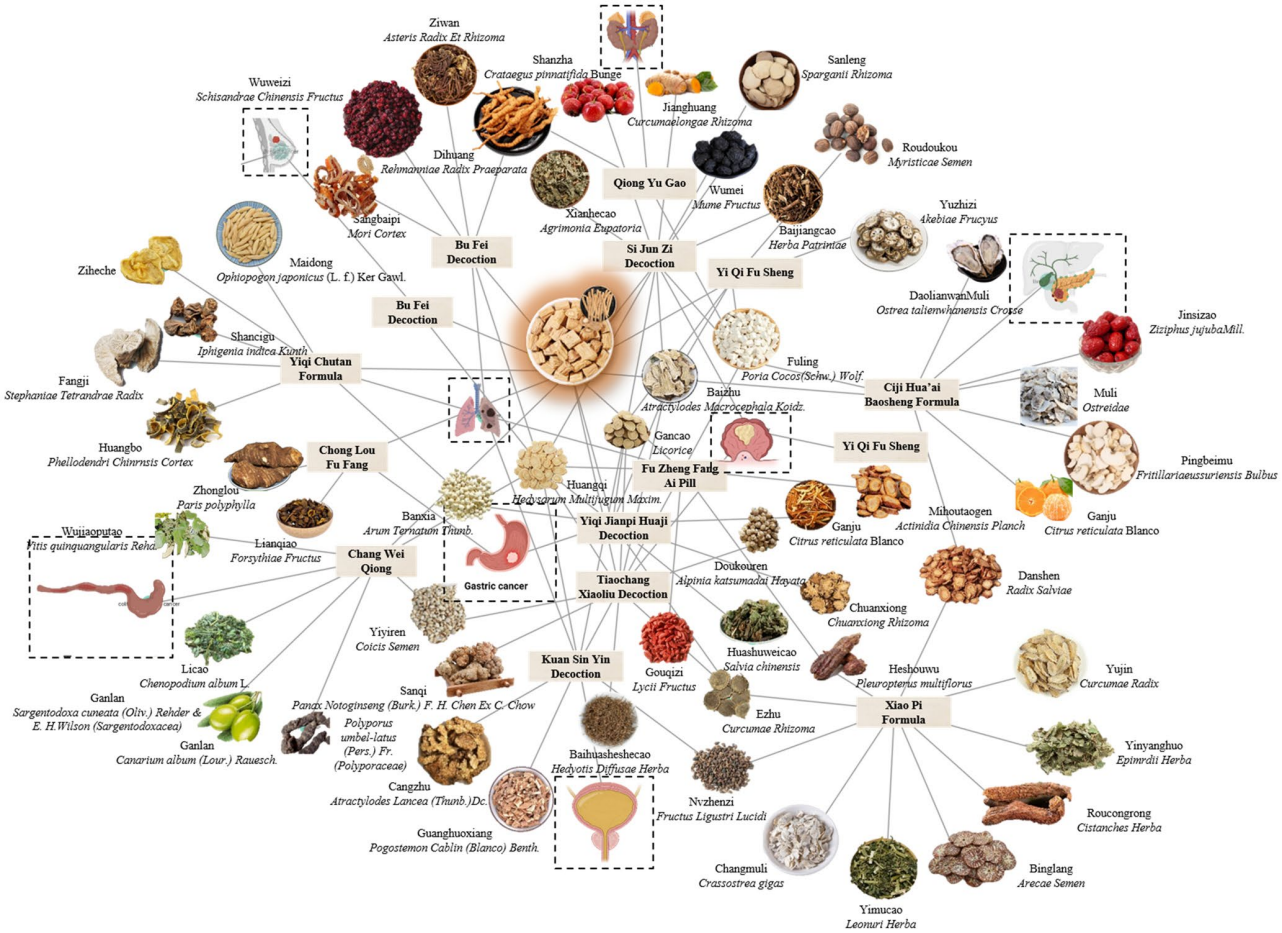
Cancers‐herbal formula‐traditional Chinese medicine network.

From the perspective of the anti‐cancer mechanism, *C. pilosula* and *C. pilosula* ‐based TCM formulations work through various aspects such as cell signal transduction, gene expression regulation, and immune‐related pathways. While all of them show inherent connections in cancer auxiliary regulation. They are all important practices of TCM as CAM, enriching the anti‐cancer methodology, complementing modern medical auxiliary regulation methods, and providing a broader perspective for comprehensive cancer auxiliary regulation. The TCM network of *C. pilosula* and *C. pilosula* ‐based herbal formula for auxiliary regulation of various cancers is shown in Figure 4. The prescriptions contribute to a deeper understanding of the mechanism of action and application value of TCM in cancer auxiliary regulation, providing a basis for further research and development of more effective anti‐cancer TCM formulations.

## 5 Further Prospects

Many researchers have contributed valuable discoveries to the scientific research of *C. pilosula* , indicating its considerable medical and nutritional value. In healthcare, its substantial medical and nutritional value could potentially lead to the development of novel drugs and health products for disease auxiliary regulation. In agriculture, further understanding can optimize cultivation techniques, enhancing yield and quality. Other industries, such as the food industry, might also develop functional foods based on *C. pilosula* . However, existing cognitive gaps in its application need to be addressed. More research is still needed to further explore the value of *C. pilosula* , including in‐depth exploration of the anti‐cancer mechanisms of *C. pilosula* and the interaction between *C. pilosula* and common anti‐cancer herbs. Well‐designed studies on the pharmacodynamic material foundation and pharmacological mechanisms should be carried out, along with comprehensive investigations to identify relevant compounds and potential mechanisms. Moreover, toxicological research of the bioactive extracts and isolated compounds from *C. pilosula* is also urgently needed to fully explore its value and bridge the application gaps.

## 6 Conclusions

TCM involves natural products that are remarkably effective and safe to use. *C. pilosula* , with a history dating back thousands of years, has had its significance highlighted due to its wide use and phytochemical investigation in TCM. This review is centered on the anti‐cancer properties, bioinformatics and biological knowledge of *C. pilosula* and related decoctions. Combining the phytochemical analysis, the bioactivities and relevant mechanisms to interpret their traditional medicinal use and advocate pharmaceutical product development will be the key for advanced research. Furthermore, *C. pilosula* may serve as a potential treasure trove for pharmaceuticals, functional foods, and cosmetic additives in the future. The available evidence warrants further research into the possible role of *C. pilosula* in preventing human carcinogenesis. In conclusion, this review provides theoretical research and reference for profound systematic testing and development in the use of *C. pilosula* in the fields of food and medicine.

## Author Contributions

**Haoran Fan:** conceptualization, data curation (equal). **Chenxi Ren:** methodology. **Yining Feng:** software (equal). **Lingyi Zhu:** data curation (equal). **Aobo Yu:** software (equal), supervision (equal). **Tianzhu Guan:** supervision (equal), writing – review and editing. All authors have read and agreed to the published version of the manuscript.

## Conflicts of Interest

The authors declare no conflicts of interest.

## Data Availability

Data are available to the corresponding author upon reasonable request.
